# Catalytic Properties of Amylolytic Enzymes Produced by* Gongronella butleri* Using Agroindustrial Residues on Solid-State Fermentation

**DOI:** 10.1155/2017/7507523

**Published:** 2017-12-10

**Authors:** Gabriéla Finoto Cavalheiro, Isadora Stranieri Sanguine, Flávia Regina da Silva Santos, Ana Carolina da Costa, Matheus Fernandes, Marcelo Fossa da Paz, Gustavo Graciano Fonseca, Rodrigo Simões Ribeiro Leite

**Affiliations:** ^1^Laboratory of Enzymology and Fermentation Processes, Faculty of Biological and Environmental Sciences, Federal University of Grande Dourados (FCBA/UFGD), Dourados, MS, Brazil; ^2^Laboratory of Bioengineering, Faculty of Biological and Environmental Sciences, Federal University of Grande Dourados (FCBA/UFGD), Dourados, MS, Brazil

## Abstract

Amylases catalyze the hydrolysis of starch, a vegetable polysaccharide abundant in nature. These enzymes can be utilized in the production of syrups, alcohol, detergent, pharmaceutical products, and animal feed formulations. The aim of this study was to optimize the production of amylases by the filamentous fungus* Gongronella butleri* by solid-state fermentation and to evaluate the catalytic properties of the obtained enzymatic extract. The highest amylase production, 63.25 U g^−1^ (or 6.32 U mL^−1^), was obtained by culturing the fungus in wheat bran with 55% of initial moisture, cultivated for 96 h at 25°C. The enzyme presented optimum activity at pH 5.0 and 55°C. The amylase produced was stable in a wide pH range (3.5–9.5) and maintained its catalytic activity for 1 h at 40°C. Furthermore, the enzymatic extract hydrolyzed starches from different vegetable sources, presenting predominant dextrinizing activity for all substrates evaluated. However, the presence of glucose was observed in a higher concentration during hydrolysis of corn starch, indicating the synergistic action of endo- and exoamylases, which enables the application of this enzymatic extract to produce syrups from different starch sources.

## 1. Introduction

Agroindustrial residues require adequate disposal as they may cause environmental problems and would be wasted if left unused, considering that these are advantageous by-products with high biomass and energy. An alternative way to use these residues is to employ them as substrates in microbial culture processes for the production of enzymes [1].

Owing to their wide availability and low commercial value, the use of these materials can contribute toward a reduction in the operational cost of enzyme production as well as toward minimizing possible environmental impacts due to their inappropriate disposal. In general, filamentous fungi are considered the most suitable microorganisms for solid-state fermentation (SSF) because their hyphae can grow on the surface of solid particles, simulating their natural habitat [2, 3].

Starch is an important constituent of the human diet and is one of the main vegetable energy storage products, found in abundance in wheat, rice, corn, cassava, and potatoes. Enzymes that catalyze the hydrolysis of starch are used in the production of maltodextrins, modified starches, glucose, and maltose syrups and can be applied in several industrial processes, such as the production of biofuels, food, pharmaceuticals, detergents, beer, bread, and animal food [4].

These enzymes can be derived from plants, animals, and microorganisms. However, fungal- and bacterial-derived enzymes are predominantly used in the industrial sector because of their lower cost and production time [5, 6]. Several microorganisms have been studied for amylolytic enzymes production, with species of* Aspergillus*,* Rhizopus*, and* Bacillus* genera being the most used in industrial processes [7–9].

Recently, our research group isolated a fungal strain with the potential for amylase production, identified as* Gongronella butleri *[10]. The development of present work was stimulated due to the limited amount of studies employing* G. butleri* for amylolytic enzymes production. In this context, this study aimed to optimize its cultivation parameters for the amylase production and to describe the catalytic properties of this enzyme.

## 2. Materials and Methods

### 2.1. Microorganism

In this study was used the mesophilic fungus* Gongronella butleri* isolated from soil samples collected from the Cerrado biome, located in Dourados, MS (Brazilian Midwest: 22°10′49.2′′S 54°56′57.4W) [10]. The microorganism was cultured at 28°C on Sabouraud dextrose agar medium and maintained at 4°C at the Laboratory of Enzymology and Fermentation Processes of the Faculty of Biological and Environmental Sciences of the Federal University of Grande Dourados (FCBA/UFGD).

### 2.2. Inoculum

The microorganism was cultivated in 250 mL Erlenmeyer flasks containing 40 mL of Sabouraud Dextrose Agar inclined and incubated for 48 h at 28°C. A fungal suspension was obtained by adding 25 mL of nutrient solution and gently scraping the surface of the culture. The nutrient solution was composed of 0.1% ammonium sulfate, 0.1% magnesium sulfate heptahydrate, and 0.1% ammonium nitrate (w/v).* G. butleri* was inoculated by transferring 5 mL of the microbial suspension to each 250 mL Erlenmeyer flask containing agroindustrial residues (10^5^ spores/g dry substrate). The volume of the inoculum was used to calculate the initial moisture of the SSF process [10].

### 2.3. Production of Amylase by SSF

Microbial culture was performed in 250 mL Erlenmeyer flasks with 5 g of agroindustrial residues (corn straw, corn cob, rice peel, soy bran, and wheat bran) moistened with the above-mentioned nutrient solution. All substrates were washed with distilled water and dried in an oven at 50°C for 48 h. Prior to inoculation of the microorganism, all materials used were autoclaved for 20 min. at 120°C. The substrates were inoculated with* G. butleri*, with the initial moisture of 65%, and incubated at 30°C for 96 h. The substrate presenting with best enzyme production was used to evaluate other fermentative parameters, such as moisture (50–75%), temperature (20–40°C), and incubation period (24–120 h), and the optimal condition of each experiment was adopted in subsequent trials. All assays were carried out in triplicate and the obtained results represented by their respective averages and standard deviations [1].

### 2.4. Enzyme Extraction

Enzyme extraction was performed by adding 50 mL of distilled water in the fermented substrate with continuous agitation at 100 rpm for 1 h. The samples were filtered through nylon cloth and centrifuged at 1500 ×g for 5 min. at 5°C. The supernatant was considered the enzymatic extract and was used in subsequent assays [10].

### 2.5. Determination of Amylase Activity

Amylase activity was determined by adding 0.1 mL of enzymatic extract in 0.9 mL of sodium acetate buffer (0.1 M, pH 5.0), containing 1% corn starch. The reaction mixture was incubated at 50°C for 10 min. The reducing sugars released were quantified by measuring the absorbance at 540 nm by the DNS method (3,5-dinitrosalicylic acid) [11]. A unit of enzyme activity was defined as the amount of enzyme required to release 1 *μ*mol of product per minute of reaction.

### 2.6. Biochemical Characterization of Amylase Produced

#### 2.6.1. Effect of pH and Temperature on Enzymatic Activity

Optimum pH was determined by measuring enzyme activity at 50°C at different pH values (3.0–8.0) using the McIlvaine buffer. Optimum temperature was determined by the dosage of the enzymatic activity under different temperature conditions (30–75°C), at the respective optimum pH of the enzyme. Stability of the enzyme at different pH was evaluated by incubating the enzyme with different pH values for 24 h at 25°C. The buffers used were McIlvaine (pH 3.0–8.0), 0.1 M Tris-HCl (pH 8.0–8.5), and 0.1 M Glycine–NaOH (pH 8.5–10.5). Thermostability was studied by incubating the enzyme for 1 h under different temperature conditions (30–75°C). Residual activities of the enzyme were determined under optimum pH and temperature [4].

#### 2.6.2. Evaluation of the Catalytic Potential for Several Starch Sources

The enzymatic extract was evaluated for its catalytic potential for hydrolyzing starch from several vegetable sources. The enzymatic assays were performed using 1% potato, wheat, cassava, lentils, beans, rice, oats, sweet potato, and corn starch as the substrates. The experiment was performed in sodium acetate buffer (0.1 M, pH 5.0) at 50°C for 10 min., and the released reducing sugar was quantified by the DNS method [11].

#### 2.6.3. Evaluation of the Dextrinizing Potential of the Enzymatic Extract

Dextrinizing activity was evaluated using starches highly susceptible to the enzymatic action. The assays were conducted in sodium acetate buffer (0.1 M, pH 5.0) containing 1% starch, and depolymerization of starch was quantified by the iodometric method described by Fuwa [12] and Pongsawadi and Yagisawa [13]. The reaction mixture was composed of 0.1 mL of amylase and 0.3 mL of starch-containing buffer. After incubation for 10 min at 55°C, the reaction was stopped by the adding 4 mL of 0.2 M HCl, 0.5 mL of iodine reagent (0.3% KI and 0.03% I_2_), and 10 mL of distilled water. Absorbance was measured at 700 nm. One unit of enzyme activity was defined as the amount of enzyme required to reduce the intensity of the blue color of the iodo–starch complex by 10% per minute of reaction.

#### 2.6.4. Saccharifying Potential of the Enzymatic Extract

The saccharifying activity was evaluated using starches that presented greater susceptibility to the enzymatic action. Assays were performed in sodium acetate buffer (0.1 M, pH 5.0) containing 1% starch. The glucose released was quantified by the glucose oxidase/peroxidase method described by Bergmeyer and Bernt [14]. The reaction mixture was composed of 0.1 mL of amylase and 0.4 mL of starch-containing buffer solution. After incubation for 10 min at 55°C, the reaction was stopped in ice bath. The glucose released was quantified with the enzymatic colorimetric kit (Glucose-PP Analisa). The absorbance was measured at 505 nm. One unit of enzyme activity was defined as the amount of enzyme required to release 1 *μ*mol of glucose per minute of reaction.

#### 2.6.5. Chromatography of Hydrolysis Products

Chromatographic analyses of the reaction products of amylase activity on soluble starch were performed using thin layer chromatography. A volume of 0.01 mL of the reaction mixture was applied on silica gel plates (G-60, 10 × 15 cm) and subjected to two sequential ascending chromatography runs using butanol/ethanol/water (5 : 3 : 2) as the solvent system. After air drying the plate, spots developed by spraying with a solution of H_2_SO_4_ and methanol (1 : 9) containing 0.2% orcinol, and heating at 100°C [15].

### 2.7. Statistical Analysis

All experiments were carried out as triplicate, and the results are presented as the mean of three independent assays and their standard deviations. Statistical analyses of the data included a one-way ANOVA followed by Tukey's test with 1% significance.

## 3. Results and Discussion

### 3.1. Amylase Production by SSF

Among all the substrates evaluated in the present study, cultivation on wheat bran resulted in higher amylase production by the fungus* G. butleri*, approximately 35.57 U g^−1^ of dry substrate (Table 1).

Most filamentous fungi when cultivated in solid state exhibit higher enzymatic production on wheat bran due to their nutritional properties, aeration, and efficient penetration of the mycelia [16]. Chimata et al. [17] reported higher amylase production by different* Aspergillus *species using wheat bran as a substrate on SSF.

Previous studies have confirmed wheat bran as an excellent substrate for the cultivation of filamentous fungi to produce several enzymes, such as amylases, *β*-glucosidases, xylanases, and cellulases [1, 4, 10, 18]. Thus, wheat bran was selected as the substrate for subsequent assays.

**Figure 1:**
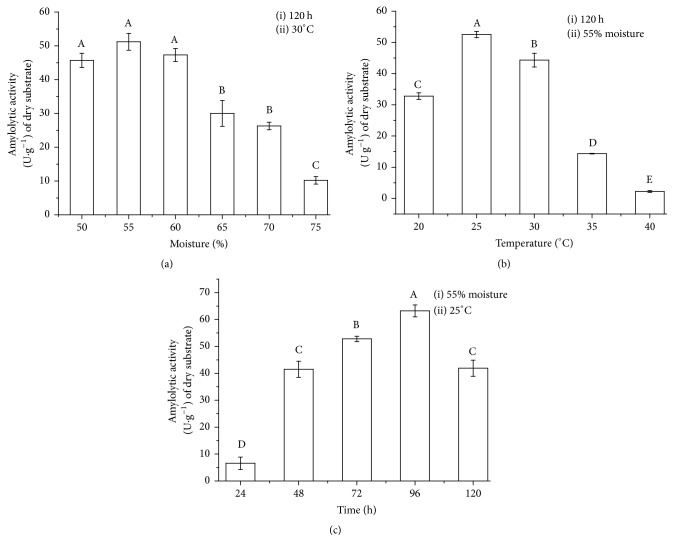
Production of amylase by solid-state fermentation by the fungus* Gongronella butleri* in wheat bran. (a) Initial moisture, (b) culture temperature, and (c) culture time. Average productions with different letters indicate significant differences (*p* < 0.01) according to Tukey's test.

Optimal moisture for amylase production by* G. butleri* was between 50 and 60%. The highest production of amylase in absolute values was 51.26 U g^−1^ and was obtained in cultures with 55% moisture; thus, this value was adopted as the optimum moisture content in subsequent assays. However, no significant difference was observed between the cultures with 50, 55, and 60% of initial moisture (Figure 1(a)).

Moisture content of the substrate is one of the factors that influences microbial culture in the solid state. Water diffuses the constituents of the culture medium, thereby dispersing metabolites and leading to effective absorption of nutrients by microorganisms. Availability of water in the culture medium favors biological functions and structurally stabilizes biomolecules. However, reduced water activity can affect the process of germination, sporulation, and formation of metabolites, which compromises enzymatic production [1, 18].

Several temperatures were evaluated for SSF aiming at the production of amylolytic enzymes. Higher production of amylase, 52.54 U g^−1^ of dry substrate, was obtained in cultures maintained at 25°C, and at temperatures above 30°C, reduction in enzymatic production was observed (Figure 1(b)), a profile typically observed in mesophilic microorganisms [10]. The influence of temperature on the production of enzymes is directly related to the growth of the microorganism. Most of the mesophilic strains used to produce amylases exhibit higher enzymatic production when cultured at 25–35°C [19].

One of the biggest challenges of the SSF process is metabolic heat dissipation generated by microbial growth, as very high temperatures can cause membrane collapse and denature structural proteins and enzymes; however very low temperatures reduce the permeability of the plasma membrane and the speed of metabolic reactions [1]. Therefore, evaluation of culture temperature is crucial in SSF [2].

To optimize the culture time for amylase production, samples were removed every 24 h for a total of 168 h. Highest production of amylases was obtained with 96 h of cultivation, approximately 63.25 U g^−1^, with other optimal parameters, as obtained in previous assays (Figure 1(c)).

The cultivation time for amylase production, obtained in the present study, was similar to or less than that described for the production of amylases by other fungal strains. Kunamneni et al. [20] reported higher amylase production in 120 h of culture by* Thermomyces lanuginosus *on SSF using wheat bran as substrate. Furthermore, Ahmed [21] described higher production of amylase by* Aspergillus oryzae* in 120 h of SSF. Therefore, the reduced cultivation time is an essential characteristic for the production of enzymes of industrial interest because it directly influences the cost of the process [1, 18].

The results obtained in the present study confirm the potential for the production of amylolytic enzymes by* G. butleri* in low-cost media, particularly when compared with other fungal species previously described for the production of amylases. Ferreira et al. [22] reported the production of 16.58 U g^−1^ by* Chrysosporium zonatum* in 144 h and 55.06 U g^−1^ by* Malbranchea pulchella* in 144 h of SSF. The microorganism* Rhizopus oryzae* presented a production of 63.50 U g^−1^ when cultured for 24 h on wheat bran [23]. Bernardes et al. [24] reported production of 13 U g^−1^ of amylase by* Rhizomucor miehei *in 48 h of cultivation.

### 3.2. Biochemical Characterization of Amylase

#### 3.2.1. Effect of pH and Temperature

**Figure 2:**
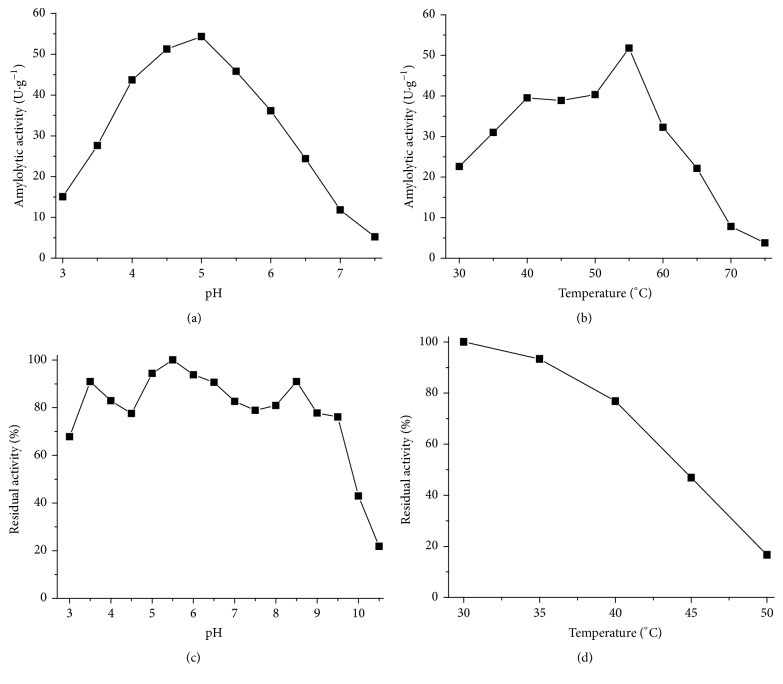
Biochemical characterization of amylase produced by solid-state fermentation of wheat bran by* Gongronella butleri*. (a) Optimum pH, (b) optimum temperature, (c) stability pH, and (d) stability temperature.

Amylase produced by* G. butleri* presented optimal activity at pH 5.0 (Figure 2(a)). However, high enzymatic activity was observed in the pH range of 4.0–5.5. Fungal amylases described in literature present optimum activity at different pH values, with the pH commonly between 4.5 and 7.0 [25]. Negi and Banerjee [26] describe 4.0 as the optimal pH for amylase production by* Aspergillus awamori*, whereas amylase produced by* Lichtheimia ramosa* presented higher catalytic activity at pH 6.0 [4].

Amylase evaluated in the present study presented optimum activity at elevated temperatures (55°C) when compared to amylases produced by other fungal species (Figure 2(b)). Soni et al. [27] reported higher catalytic activity of the glucoamylase of* Aspergillus *sp. at 50°C. The optimum temperature demonstrated for amylase produced by* Fusarium solani* was 40°C [28]. Kunamneni et al. [20] described 50°C as the optimal temperature for amylase production by* Thermomyces lanuginosus*.

The amylase produced by* G. butleri* presented high catalytic activity over a wide range of pH and temperature (Figures 2(a) and 2(b)). This characteristic is extremely interesting for the industrial application of this enzyme, considering that the pH and temperature controls in industrial processes are less efficient compared to laboratory conditions [1].

With respect to pH stability, the enzyme retained more than 78% of the original activity after being incubated in a wide pH range (3.5–9.5) for 24 h (Figure 2(c)). The variation of the enzymatic stability observed throughout the analyzed pH range may be an indicative of the presence of different amylolytic complex enzymes in the enzymatic extract composition, considering that the assays were performed with crude enzymatic extract (Figure 2(c)).

The high pH stability of amylase obtained in this study becomes even more evident when compared with enzymes of other fungal species. Nwagu and Okolo [29] reported that amylase produced by* Aspergillus fumigatus *maintained 94% of its activity at pH 4.5–6.5 for 24 h, and amylase produced by* Syncephalastrum racemosum *remained stable for 24 h between pH 4.0 and 8.0 [30].

Amylase produced by* G. butleri* retained 80% of its catalytic activity after 1 h at 40°C. When incubated for the same period at 45°C, the enzyme lost about 50% of the initial activity (Figure 2(d)). This characteristic is very important for industrial application, as most processes require thermotolerant enzymes. *α*-Amylase produced by* Aspergillus oryzae* is reported to retain only 70% of its original activity after 1 h at 28°C [31]. Adeniran and Abiose [32] described the thermostability of amyloglucosidase produced by* Aspergillus niger*; this enzyme maintained 60% of its catalytic activity at 35°C for 1 h, and when incubated for the same period at 40°C, only 50% of initial activity was recovered.

In general, enzymes secreted into the extracellular medium have greater structural stability compared to intracellular enzymes, especially when produced by solid-state fermentation [2]. Most extracellular enzymes have carbohydrates attached to their structure (e.g., glycoproteins), which confer greater structural stability [33]. However, protein stability cannot be attributed solely to glycosylation. Different types of bonds (covalent or not) contribute to maintaining the tertiary structure of a protein, for example, hydrophobic, electrostatic, ionic, hydrogen, and disulfide interactions. Thus, small variations in the structural composition of a protein may result in greater or lesser stability to physical and chemical agents [4].

#### 3.2.2. Evaluation of Catalytic Potential for Several Sources of Starch

**Figure 3:**
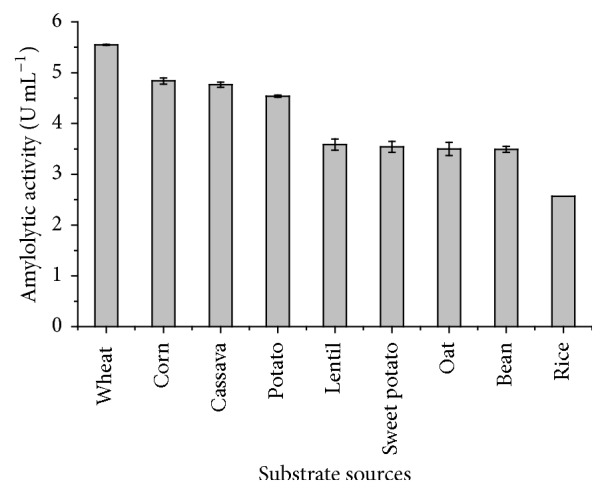
Evaluation of the catalytic potential of the amylase produced by* Gongronella butleri* on starch from different vegetable sources using the 3,5-dinitrosalicylic acid (DNS) method, used to quantify total reducing sugar.

The enzymatic extract was evaluated for the potential of hydrolyzing starches from several vegetable sources. Starches from corn, wheat, rice, beans, oats, lentil, potato, and sweet potato were used in this study. Amylase obtained in this study degraded all the starches tested (Figure 3). This characteristic enables the use of this enzyme to hydrolyze starches from different sources. Catalytic equivalence for starches from different vegetable sources is not commonly observed, considering that starches from different botanical sources have distinct structural characteristics, which can interfere in enzymatic catalysis [34, 35].

According to Fennema et al. [36], the higher the level of branching in the starch structure (e.g., amylopectin), the lower the catalytic efficiency of the amylolytic enzymes. Oliveira et al. [35] compared the catalytic potential of amylases produced by different yeast species in starches extracted from corn, wheat, potato, and cassava. The authors reported higher efficiency of the enzymes to hydrolyze corn starch and justified the results by stating lower concentration of amylopectin present in its structure.

Similar results were not obtained in the present study. The potential of hydrolyzing different types of starches, observed for the enzyme produced by* G. butleri*, may be associated with the synergistic action of debranching enzymes (e.g., isoamylases), which disorganize amylopectin and favor the performance of endo- and exoamylases. The results of pH stability discussed here indicate that the enzymatic extract produced by* G. butleri* does not present only one enzyme of the amylolytic complex.

#### 3.2.3. Evaluation of the Dextrinizing and Saccharifying Potential of the Enzymatic Extract

**Figure 4:**
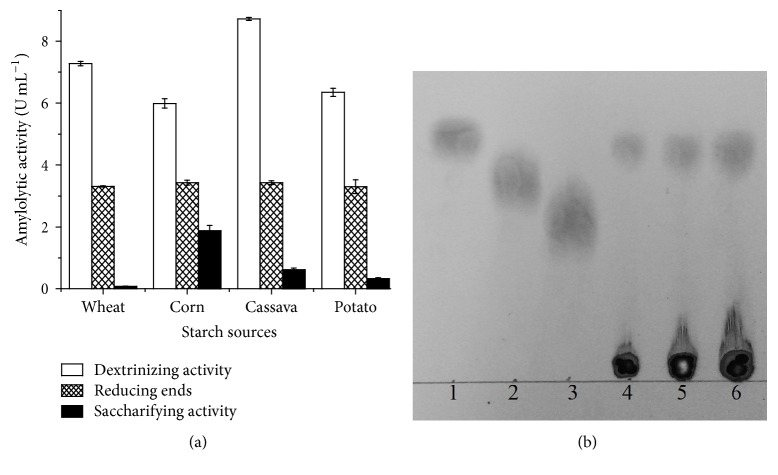
(a) Enzymatic modifications on starch from wheat, corn, cassava, and potato by colorimetric methods and (b) thin layer chromatography of the corn starch hydrolysate. Standards: (1) glucose; (2) maltose; (3) maltotriose. Hydrolyzed at different times: (4) 10 min; (5) 15 min; (6) 20 min.

Amylase produced by* G. butleri* presented predominantly dextrinizing activity, owing to higher potential for depolymerization of the starch, as evidenced by the iodometric method. However, different amounts of glucose were recovered in the hydrolysates after the enzymatic treatment, as quantified by the glucose oxidase/peroxidase method and confirmed by thin layer chromatography (TLC) (Figures 4(a) and 4(b)).

The enzymes produced by* G. butleri* reduced starch polymerization degree, resulting in high amount of reducing chain ends and the release of glucose monomers, showing a higher concentration of monosaccharides in the assays with corn starch (Figures 4(a) and 4(b)).

The distinct efficiency in the conversion of starch to glucose observed in this study is possibly related to the structure of the polysaccharide used as substrate (Figure 4(a)). As previously described, starches from different vegetable sources have different structural composition [34]. Corn starch contains less branching, favoring the action of amylolytic enzymes [35], which justifies higher saccharification of this polysaccharide in comparison with others evaluated in the present study.

Therefore, the enzymatic extract produced by* G. butleri* presents predominantly dextrinizing activity (endoamylases) with reduced saccharifying potential (exoamylases). Previous studies have confirmed majority production of dextrinizing enzymes by different fungal species. Shafique et al. [37], Cruz et al. [38], Sahnoun et al. [39], and Pervez et al. [40] reported endoamylases activity for different species of* Aspergillus*, such as* A. flavus*,* A. niger*,* A. oryzae*, and* A. fumigatus*, respectively.

## 4. Conclusions

The obtained results allow inferring that the filamentous fungus* G. butleri* presents potential for the production of amylases in low-cost culture media (agroindustrial residues), with relatively less production time. The enzyme produced in this study exhibited interesting characteristics for industrial application, such as activity and stability in a wide range of pH and temperature. Furthermore, the enzyme exhibited excellent ability to degrade different starches with predominant dextrinizing activity. Another important aspect that should be emphasized is the reduced number of studies using* G. butleri* for the production of industrial enzymes, which encourages the development of new works with this strain.

## Figures and Tables

**Table 1:** Production of amylase by the fungus *Gongronella butleri* in different agroindustrial residues containing 65% moisture for 120 h of cultivation at 30°C.

Substrates (agroindustrial residues)	Amylase (U g^−1^ of dry substrate)
Corn straw	6.03 ± 0.63^c^
Corn cob	2.29 ± 0.5^d^
Rice peel	2.71 ± 0.4^d^
Soy bran	9.56 ± 0.46^b^
Wheat bran	35.54 ± 1.01^a^

^a, b, c, d^Average productions with different letters indicate significant differences (*p* < 0.01) according to Tukey's test.
